# Screening for social difficulties in cancer patients: clinical utility of the Social Difficulties Inventory

**DOI:** 10.1038/sj.bjc.6604006

**Published:** 2007-09-25

**Authors:** P Wright, A Smith, K Roberts, P Selby, G Velikova

**Affiliations:** 1 Psychosocial and Clinical Practice Research Group, Cancer Research UK, Clinical Centre in Leeds, St James's University Hospital, Leeds LS9 7TF, UK; 2 Leeds City Council Social Services Department, Cookridge Hospital, Leeds LS16 6QB, UK

**Keywords:** social difficulties, patient-centred assessment, psychosocial, oncology

## Abstract

Guidelines for psychosocial support have been developed, but there are no standard approaches in routine oncology practice to identify patients experiencing social difficulties. We have designed and evaluated a Social Difficulties Inventory (SDI) to identify patients requiring further assessment and, where appropriate, referral to support services. The purpose of this study was to develop a clinically meaningful SDI scoring system with guidance for oncology staff. Out of 189 patients, 183 completed the SDI and were interviewed by a social work researcher who scored the SDI independently. Comparison of patient/interviewer assessment was good (intraclass correlation 0.61, 95% confidence interval: 0.51, 0.70). Using top 10% of interviewer social distress (SD) scores to indicate ‘SD case’, the best ‘cut-point’ was a patient score of ⩾10 (sensitivity=0.80; specificity=0.76; 56 out of 183 ‘cases’). Out of 127 patients, 72 with SD score <10 had individual SDI item rated at a higher level. Following interview, 32 patients were referred to specialist services, 46 given information and 112 had no action taken. An interpretation algorithm developed includes SD score, individual SDI item rating, and an additional general question, illustrated using four case scenarios. In conclusion, general guidance for interpreting the SDI has been developed to enhance health-care professional/patient consultations with a view to identifying patients who may benefit from support, advice or intervention.

## 

A diagnosis and treatment for cancer impacts on the everyday lives of patients: at home, work and leisure resulting in a range of social changes that may be problematic (Carelle *et al*, 2002; Wright *et al*, 2002). Issues may be resolved by patients with no reference to anyone outside their circle of family and friends (Eakin and Strycker, 2001). However, others may benefit from information or assistance from health- and social- care professionals. There has been a steer to integrating psychosocial patient-centred assessment into routine oncology practice with national bodies developing guidance for implementation (Canadian Association of Psychosocial Oncology, 1999; National Comprehensive Cancer Network, 1999; National Breast Cancer Centre and National Cancer Control Initiative, 2003). UK guidance from the National Institute for Clinical Excellence (NICE) recommends routine assessment of social support covering emotional support, help with personal care, employment and financial advice, help at home, practical aids and adaptations and help to care for dependents (National Institute for Clinical Excellence, 2004). This would allow provision of psychosocial information, supportive care or more complex interventions to be directed appropriately dependent on the level of distress identified and following discussion with the patient.

Little information is available on the proportion of referrals to psychosocial services from oncology outpatients. Locally in 2006, 3.2% of Cookridge Hospital outpatients (a specialist oncology hospital) were referred to social work and 1.5% of outpatients from across the Leeds Cancer Centre to the psychosocial team (liaison psychiatry, clinical and health psychology). Referral rates may be low due to a number of factors, including lack of training in communications skills (Fallowfield and Jenkins, 1999), reluctance on the part of patients to take up offers of support (Roth *et al*, 1998) or uncertainty about whose roles to provide this assessment it is within the multidisciplinary team (Catt *et al*, 2005). Poor identification of problems may be a contributory factor to referral rates (Cull *et al*, 1995). Introducing routine assessment, using standardised questionnaires, may provide a better way of identifying patients with problems who might benefit from discussion and possible referral. As cancer clinics are busy, social problems assessment would have to meet requirements of brevity, simplicity, relevance, practical utility and ease of scoring. A number of oncology-specific questionnaires have been developed encompassing aspects of social assessment including the 59-item Cancer Rehabilitation Evaluation System short form (CARES-SF) (Schag and Heinrich, 1991), the 61-item Supportive Care Needs Survey (Bonevski *et al*, 2000) and the Distress Thermometer, a single-item self-report measure of distress accompanied by a list of 34 problems (Roth *et al*, 1998; Jacobsen *et al*, 2005). In a review of cancer patients’ needs assessment tools, no one instrument was identified that covered the social domain comprehensively (Richardson *et al*, 2005).

As part of a research programme aimed at developing and evaluating a system of routine patient-centred assessment, we have created and validated a 21-item questionnaire, the Social Difficulties Inventory (SDI), to assess social difficulties experienced by cancer patients (Table 1) (Wright *et al*, 2002, 2005; Smith *et al*, 2007). Validation analyses of the SDI suggested two ways to employ the SDI in clinical practice: (1) using the responses to the 21 individual items to ‘flag’ items of concern to be discussed with the patient and (2) using an overall score of ‘social distress’ (SD) to identify patients with multiple problems requiring assessment. The SD score, derived using Rasch analysis of the instrument, revealed that 16 of 21 items formed a unidimensional scale, named social distress (SD), accounting for 72% of the variance. The SD scale functions equally well irrespective of the extent of disease, disease site, gender, age and level of deprivation. Differences in scores are equally spaced, creating an interval scale over almost the entire range of raw scores and allowing the responses from the 16 items to be summed to produce score of SD. The five items not fitting the Rasch model remain within the SDI but do not contribute to the SD score (Smith *et al*, 2007) (see Table 1 for the SDI and SD scoring system).

Before recommending SDI for routine use, clinical meaning of the overall SD score and individual items must be evaluated and guidelines developed for professional use.

### Aim

The aims of the study were to (1) examine the clinical meaning and utility of the SDI by comparing patient's self-assessment with social worker assessment; (2) derive a cutoff level for overall SD scores, identifying socially distressed patients (‘case’ identification); and (3) produce initial guidance for clinic use on how to interpret the SDI item and SD scores.

## MATERIALS AND METHODS

Following local ethical approval, a cross-sectional interview study was undertaken in Leeds, a tertiary cancer centre, between January 2003 and May 2004. Patient eligibility included the ability to read English, physical and mental capability to complete questionnaires via a computer touchscreen and non-participation in other psychosocial oncology studies.

### Patients

Adult patients, approached consecutively, were recruited from outpatient clinics or day units in haematology, medical oncology, clinical oncology and chest medicine. Sociodemographic and clinical data were collected on those who declined participation.

### Questionnaires

Patients completed four questionnaires using a computer touchscreen in clinic (1) SDI (Wright *et al*, 2005), (2) Hospital Anxiety and Depression Scale (HADS) (Zigmond and Snaith, 1983), (3) European Organisation for Research and Treatment of Cancer Quality of Life Questionnaire (EORTC QLQ-C30) (Aaronson *et al*, 1993) and (4) Close Persons Questionnaire (CPQ) (Stansfeld and Marmot, 1992). In addition, they indicated whether they thought they would have benefited from help over the last month for any SDI items. In this paper, only SDI results are reported.

Patients provided sociodemographic information and clinical data were collected from medical notes (age, gender, postcode, diagnosis, date of diagnosis and disease stage). Deprivation levels were determined using Indices of Multiple Deprivation (IMD) based on postcodes and derived from 2001 UK census (Office for National Statistics, 2001; Noble *et al*, 2004).

### Interviews

Within a week of the touchscreen assessment, participants were interviewed at home by PW, a social worker investigator, who was blind to the results from the touchscreen assessment. The interview was semistructured, lasted for about an hour and was audio-recorded. The interview concerned the last month and began with a general overview of the patient's cancer and cancer treatment followed by more detailed questioning on each of the domains of the SDI: ‘physical ability’, ‘providing for others’ and ‘contact with others’ and the single items, derived from the factor analysis undertaken in the psychometrics study (Wright *et al*, 2002). The nature of any difficulties and their resolution was explored and additional questions asked dependent upon responses provided by the participant. On the completion of the interview, any items of concern raised by the patient were discussed and, with the agreement of the patient, either information provision or referral was arranged. On return to the office, PW completed the SDI according to the content of the interview.

An independent oncology social worker (KR) listened to a random 10% of recordings and provided SDI scores (Wimmer and Dominick, 2006; p 167). The sample comprised of 10 women and 9 men with an age range of 40–75 years and from a number of different diagnostic groups. The length of interviews ranged from 20 to 95 min. Kappa (*κ*) calculation was possible for 19 out of 21 items. The majority of *κ*-values were >0.60 (good to very good agreement), five were above 0.40 and five above 0.20 (fair agreement).

### Statistical analyses

Differences between participants and non-participants were checked using *χ*^2^ test and *t*-tests.

#### Comparison between patient-reported and investigator's SDI scores

Patient-reported SDI scores were compared to the investigator-assigned SDI scores. Measures of agreement (*κ*-values) and association (Spearman's correlation coefficients) were calculated for individual items. Patient's and investigator's overall SD scores were compared using paired sample *t*-test and intraclass correlation (ICC).

#### Deriving a cutoff score for case identification

The top 10% of investigator-identified overall SD scores were taken as the best available indicator of caseness of SD. The overall SD score at the cut-point between the 10% distressed and 90% non-distressed was noted. The investigator's opinion on patient SD ‘caseness’ (yes/no) was treated as the definitive decision. A receiver-operating characteristic (ROC) curve was calculated on the overall ‘SD’ patient scores to derive a cut-point for guidance on score interpretation for clinical use. Sensitivity (true cases/true cases plus false positives), specificity (true normals/false positives plus true normals) and positive predictive values (true cases identified/false positives plus true cases correctly identified) for the best cut-point were calculated.

### Developing guidance for using the SDI in clinical practice

To develop practical guidance, SDI results were linked to subsequent interventions by the investigator. Patients who scored above the cutoff on overall SD score or ‘2 – quite a bit’ or ‘3 – a lot’ of difficulty on individual items were identified. The interventions made after the interview (blinded to patient's SDI results) were recorded and classified (provision of information, referral to support services). An algorithm for identifying cases is suggested and examples provided using these comparisons.

## RESULTS

### Participants and non-participants

One hundred and ninety-one patients of 273 approached consented to participate (70%). Patients in the refusing group were older than participants (*t*=−2.251, d.f.=271, *P*=0.025). The refusing group tended to be less affluent than the consenting group, but this did not reach statistical significance; IMD participants range 2.01 (most affluent) to 75.85 (most deprived), median score=16.76 and non-participants range 2.58 (most affluent) to 73.21 (most deprived), median score=20.29 (*t*=−1.931, d.f.=271, *P*=0.055). There were no differences found in terms of gender (*χ*^2^=3.369, d.f.=1, *P*=0.066).

Two participants did not complete the touchscreen assessment and six interviews were cancelled by patients, resulting in 183 full participants. Sociodemographic and clinical variables are presented in Table 2. Three participants were from ethnic minority groups.

#### Comparison between patient-reported and investigator's SDI scores

*Item by item:* There was a significant association between SDI item scores of patients and investigator (Spearman's correlation coefficient range: 0.186–0.728), although exact agreement was poor to moderate (*κ* coefficients range: 0.058–0.495) due to investigator's underestimation of difficulty.

*Overall SD score:* Using the 16-item scoring system (Table 1), SD was calculated for all patient and social work investigator assessments. Patient's and investigator's SD scores were significantly correlated (ICC=0.61, 95% confidence interval: 0.51–0.70). Investigator's SD (mean=6.96, s.d.=4.928) was lower than patient's SD (mean=7.93, s.d.=6.344) (*t*=−2.637, d.f.=182, *P*=0.009), although the *η*^2^ statistic (0.037) indicated only a small to moderate effect size.

**Figure 1:**
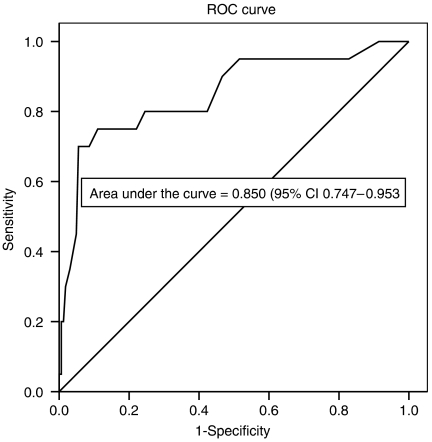
ROC curve comparing patient's SD scores with investigator-defined distress.

*Deriving a cutoff score for case identification:* Investigator's SD scores ranged from 0 to 21 (from possible range 0–44) with the top 10% of scores within the range 14–21. Using an investigator score of 14 and above as an indicator of SD caseness, the best possible cut-point for case detection derived from the ROC curve (Figure 1) was a patient score of 10 and above: sensitivity 80%, specificity 75% and positive predictive value 29%.

### Developing guidance for using the SDI in clinical practice

**Figure 2:**
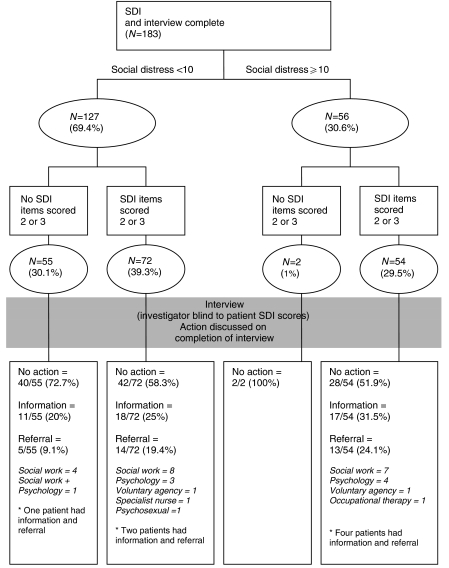
Flow chart linking patient's self-reported SDI scores with social worker's interventions, following the interviews.

Figure 2 provides a flow chart linking patient's self-reported SDI scores with social worker interventions, following interview. The number of SD cases identified using the score of 10 as the cut-point was 56 (30.6%). Two-thirds of patients (126 out of 183) rated at least one item at ‘2 – quite a bit of difficulty’ or ‘3 – very much difficulty’. Using these methods in combination, irrespective of item content and other clinical considerations, this would lead to 128 out of 183 (69.9%) patients warranting further discussion in clinic.

**Figure 3:**
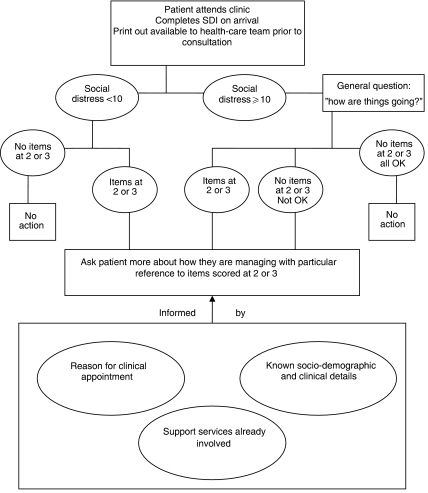
Algorithm for identification of patients who may benefit from discussion of social difficulties.

One hundred and twelve patients (61.2%) received no intervention following the patient interview (Figure 2). Referral and/or information were provided for the remaining 71 participants, ranging from simple leaflet information to referral to social work for complex problems. Referral rates were 24.1% for patients above the SD cut-point with items rated at ‘2’ or ‘3’. However, referral was also made for 5% of those with low SD (<10) with no items rated at ‘2’ or ‘3’. It would be inappropriate to recommend a prescriptive guidance for referral based on SDI results only. Therefore, we propose an algorithm for identifying patients with social problems, based on SDI results and cutoff level for SD score and also taking into account other information known about the patient, the type of clinical appointment and existing support services involved (Figure 3).

#### Guidance for using the SDI in routine clinical practice

The general guidance for the four groups of patients classified according to SDI results is described below. Specific cases illustrating the use of the guidance are provided in Table 3.

*(1) Socially distressed: patients scoring 10 or more on the overall SD scale.* These patients warrant a general enquiry from health professionals about how they are managing, with reference to high-scoring items. If the high-scoring difficulties are likely to be transitory, fade with clinical resolution or are being addressed by ongoing support, limited action may be taken (Example 1).

Patients with a number of high-scoring items, especially items less commonly endorsed, unexpected given other circumstances or in combination suggest a complex picture, should be asked more about these difficulties (Example 2).

*(2) Not socially distressed: patients scoring under 10 on the SD scale.* These patients would not automatically be followed up with a general enquiry about how they are managing. If most or all items were at the lower levels or if, in the experience of the member of staff, an item rated at a higher level is likely to be transitory or is being addressed by ongoing support then limited or no action is indicated (Example 3).

If any of the individual items were endorsed with scores of 2 or 3, this may indicate the need for further enquiry addressing those items (Example 4).

## DISCUSSION

Close association was found between patient's self-report and investigator's rating of SDI and SD, although investigator ratings were lower than patients. The fact that social workers underestimate SD reported by patients is perhaps not surprising. Social workers tend to have people referred to them who are *in extremis* with complex family or financial problems. This means their ‘benchmark’ may be at a higher starting point than the average patient. In addition, they may pay more attention to problems for which they have a remedial action. Other health-care professionals, for example, nurses or doctors, may have had a different interpretation of the social issues elicited during interview. In a study comparing quality of life (QL) scores over 12 domains, physicians underestimated the severity of patient's experience in 10 of the symptom/functioning scales (Petersen *et al*, 2006). Of the two scales overestimated by physicians, the social functioning scale showed greatest disagreement, possibly reflecting the area physicians tend to have least experience of from their clinical practice.

Using novel methodology, a cutoff for SD was derived, an initial algorithm was developed and guidance for using SDI in clinical practice produced.

For patient-centred assessment to have clinical value, instruments must be evaluated beyond basic psychometric properties. This has been achieved in psychiatry. Instruments such as the HADS have ‘cutoff’ levels for case level anxiety or depression detection using DSM systems derived from interviews (Razavi *et al*, 1990). There is no equivalent system for calibrating social difficulties, and therefore the decision to take the top 10% of SDI scores, as identified by the social work investigator, was made as the best available ‘gold standard’. This is a study limitation made for pragmatic reasons. However, the area under the ROC curve, derived using this methodology was 0.85, a level generally thought of as being in excess of acceptable levels required for screening and validated by good inter-rater reliability demonstrated between social work investigator and oncology social worker.

Rasch analysis of the SDI has provided the basis to derive a cut-point using this ‘gold standard’. As there is no differential item functioning within the Rasch SD scale for age (Smith *et al*, 2007), the fact that older patients were more likely to refuse to participate should not have influenced the ‘cut-point’ calculated. The data were collected from only one cancer centre. Although provision of support services and the level of expertise of clinical staff will vary across the cancer services of UK, this is unlikely to alter the cut-point derived from Rasch due to the lack of differential item functioning. As this work progresses, we will be able to test whether item invariance holds. How health-care professionals choose to interpret the guidelines within their own settings may well be influenced by local service provision and training of staff.

There are a number of ways of utilising patient self-reported questionnaires in everyday practice. The CARES-SF has normative data available for comparative purposes. In addition, it provides the patient with the option of stating whether they would like help with items, although documentation on how this relates to item scores is limited (Coscarelli and Heinrich, 1988). Recommendation for use in clinical practice is for preliminary assessment by CARES followed by a brief interview. The Distress Thermometer provides a quick assessment of general distress with the potential for problematic items from a number of domains to be flagged (Roth *et al*, 1998; Jacobsen *et al*, 2005). Recent guidance recommends that people with a score of 4 or more should have a clinical assessment by the primary oncology team (National Comprehensive Cancer Network, 2007). Patients with flagged problems may be overlooked if their distress score falls below 4. This is particularly relevant to problems concerning childcare, insurance, transportation and work, which were not associated with reported distress (Jacobsen *et al*, 2005).

If an SD cutoff of 10 or more had been employed as the only guide for further discussion by the clinical team, 72 patients with item rates of ‘2’ or ‘3’ would have been missed of whom 30 were either referred for specialist help or provided with information. The SDI was designed to provide additional information for doctors and nurses, to highlight issues of concern and to enhance the health-care professional/patient consultations with a view to identifying patients who may benefit from support, advice or intervention. Although the SD score does provide a cut-point indicating more severe levels of SD, this should not be taken as an automatic reason for referral to social work. There will be considerable differences between patients in terms of what each regards as a severe difficulty depending upon the individuals’ personal situation; for example, not being able to get out of the house for one person may be a great restriction, whereas for someone else may be of little consequence. The SDI output is a starting point for discussion and, as with any measure, SDI scores should not be employed in isolation. Decisions to intervene, even with established tests such as X-rays, based on X-ray alone would be foolhardy as other clinical and social issues may be influential. Definitive decisions using only SDI scores would result in clinics being overwhelmed by large numbers of identified patients and staff feeling reluctant to engage with the assessment process. A balance must be found between assessment frequency, SDI results and other clinical and sociodemographic considerations to keep patient and staff burden to a minimum without losing sensitivity of identifying patients who are struggling. Earlier work has shown that younger people and those with advanced disease are particularly vulnerable to the social impact of cancer (Wright *et al*, 2002, 2005). Although sociodemographic and clinical information may not be used as a proxy for identification of social difficulties, it may be the case that clinics in which more vulnerable groups of patients attend should be prepared to have to respond to a higher level of need. This will not only require good staff training but also access to up- to -date and relevant information on services and support.

Referral rate to social work and psychology was increased three-fold in this study compared to standard practice, although the majority of participants did not require intervention. The researcher was a social worker with many years of oncology experience in the Yorkshire region. Not only did the research interview provide adequate time for eliciting problems from patients but also the interviewer was knowledgeable and confident about local support services and information available. Prior to the start of the study, discussion had taken place with the psychosocial oncology service and the oncology social work service that had both offered active support for the study. Some clinical staff may feel that they would rather not get involved in these types of discussion due to limited communication skills (Fallowfield and Jenkins, 1999) and also a lack of knowledge of local resources. Often, clinics are busy and lack privacy; staff have to undertake a number of tasks simultaneously, meaning that psychosocial assessment and referral may be neglected. Of those who received either information or referral, many could have ‘helped themselves’ if information had been available in clinic, for example, holiday and disabled parking permit information. A number of patients not experiencing problems were referred to social work for welfare benefits advice. These people, entitled to welfare benefits but unaware of their entitlement, may not have been picked up by the SDI. Again, good information displayed in outpatient clinics may overcome this inconsistency. The SDI may provide the means to identify people experiencing difficulties sooner with a simple advice from clinic staff providing easy resolution resulting in fewer patients developing complex problems requiring referral.

Not all problems require interventions; possibly a simple acknowledgement of or reference to the patient's situation may be sufficient to enhance well-being. In a study in which QL information completed by patients in a three-armed randomised trial, chronic nonspecific symptoms were discussed more frequently in consultations where patients had completed the QL assessment and this had been fed back to the physician in real time. This did not result in either longer consultations or change in patient management but did lead to a significant improvement in QL and emotional functioning (Velikova *et al*, 2004). There were a significant minority of participants who were struggling with one or more aspects of their everyday lives, who had minimal support and had not been identified in standard clinical practice. It is for these people, the SDI may play a role with the opportunity to discuss ‘flagged’ difficulties or overall SD with the clinical team leading to possible intervention. Not all interventions are complex and a simple solution may make a big difference to a patient and not be too burdensome for staff.

Having developed guidance for health-care professionals, the next step is to consider who may be best placed to respond to the SDI in clinics. Health-care teams are multidisciplinary with members having different roles and responsibilities (Matthews *et al*, 2004; Catt *et al*, 2005). It would be unrealistic to expect all team members to have expertise in responding to all items listed within the SDI. An ongoing interview study in which staff and patients are being asked about these issues should provide useful information concerning current levels of knowledge on support services, roles, responsibilities and expectations. Once this has been established, team training on score interpretation, agreed management and support services will be developed.

Future work will focus on whether or not this type of assessment applied routinely will lead to a change in management of patients or an improvement in patient's well-being.

## Figures and Tables

**Table 1:** SDI scoring for the 16-item Social Distress Summary and SDI individual items

		**Difficulty**			
**Items**	**Description**	**No**	**A little**	**Quite a bit**	**A lot**
SDI1	Independence	0	1	2	3
SDI2	Domestic chores	0	1	2	3
SDI3	Personal care	0	1	2	2
SDI4	Caring for dependents	0	1	2	2
SDI5	Support for dependents	0	1	2	3
SDI6	Welfare benefits	0	1	2	3
SDI7	Finances	0	1	2	3
SDI8	Financial services	0	1	2	2
SDI9	Work	0	1	2	2
SDI10	Planning the future	0	1	2	3
SDI11	Communicating with those close	0	1	2	3
SDI12	Communicating with others	0	1	2	3
**SDI13**	**Sexual matters**	**0**	**1**	**2**	**3**
**SDI14**	**Plans to have a family**	**0**	**1**	**2**	**3**
SDI15	Body image	0	1	2	3
SDI16	Isolation	0	1	2	3
SDI17	Getting around	0	1	2	3
**SDI18**	**Where you live**	**0**	**1**	**2**	**3**
SDI19	Recreation	0	1	2	3
**SDI20**	**Holidays**	**0**	**1**	**2**	**3**
**SDI21**	**Other**	**0**	**1**	**2**	**3**

SD=social distress; SDI=Social Difficulties Inventory.

Key: The five emboldened items (13, 14, 18, 20, 21) are not included in the scoring of SD.

To create the SD score, derived from Rasch analysis, items 3, 4, 8 and 9 rated at ‘very much (3)’ are re-rated at ‘quite a bit (2)’. The SD score is the sum of items 1, 2, 3, 4, 5, 6, 7, 8, 9, 10, 11, 12, 15, 16, 17, 19 (range: 0–44).

**Table 2:** Clinical and sociodemographic data of participants

	**Number of patients (%)**
*Gender*	
Male (median age: 60 years, range: 18–88 years)	99 (52.4)
Female (median age: 53 years, range: 23–87 years)	90 (47.6)

*Cancer site*	
Brain	1 (0.5)
Breast	30 (15.9)
Gastrointestinal	38 (20.1)
Genitourinary	14 (7.4)
Germ cell	12 (6.3)
Gynaecological	20 (10.6)
Haematology	21 (11.1)
Head and neck	11 (5.8)
Lung	24 (12.7)
Melanoma	11 (5.8)
Sarcoma	7 (3.7)

*Stage of disease*	
Disease free diagnosed <2 years	54 (28.6)
Primary local disease	36 (19.0)
Local recurrent disease	5 (2.6)
Metastatic disease	63 (33.3)
Other^a^	23 (12.2)
Disease free diagnosed >2 years (survivor)	8 (4.2)

*Marital status*	
Single	18 (9.5)
Married or cohabiting with partners	141 (74.6)
Separated or divorced	17 (9.0)
Widowed	13 (6.9)

*Who you live with*	
I live alone	23 (12.2)
I live with my partner	90 (47.6)
I live with my partner and other relatives	56 (29.5)
I live with my children	12 (6.3)
I live with other friends or relatives	8 (4.2)

*Type of accommodation*	
Owner occupied	146 (77.2)
Rented	35 (18.5)
Other	8 (4.2)

*Employment status*	
Employed (full or part time)	76 (40.2)
Retired (at retirement age or early)	75 (39.7)
Homemaker	8 (4.2)
Other (student, unemployed, other)	30 (15.9)
	
*Occupational status (for those employed only* *N*=*76)*	
Working as usual	29 (38.2)
Working more hours	4 (5.3)
Working fewer hours	19 (25.0)
Not working	24 (31.5)

a Includes people with advanced ovarian and haematological malignancies that cannot be classified using the other categories.

**Table 3:** Examples of clinical guidance

*Example 1: Socially distressed: limited action indicated*
A 55-year-old woman diagnosed with breast cancer 8 years ago. She had surgery followed by eight cycles of chemotherapy and was awaiting radiotherapy.
*SD score*=15. Individual SD items rated at 2 or 3: independence (rated 3), domestic chores (rated 2), mobility (rated 2), recreation (rated 3). Non-SD item: holidays (rated 3).
*Guidance*: ask in general how she is managing. Refer to the holiday item to check out if she wishes to discuss this. Unless she mentions any other specific concerns that in your clinical judgement you believe warrant more attention, take the issues no further as items on independence, domestic chores, mobility and recreation are likely to improve now the chemotherapy is over.
*At the interview*, she described feeling generally debilitated by the chemotherapy but was gradually improving. The couple usually shared household tasks, but now her husband was doing much more and was happy to do so. No information, advice or referral was made. Holiday issues included a conflict between the couple about a UK or foreign destination and lack of availability of holiday insurance. Information was given on insurance companies sympathetic to cancer patients and the suggestion made to discuss going on holiday with clinic staff in terms of fitness to travel.

*Example 2: Socially distressed: action indicated*
This man in his mid forties with acute myeloid leukaemia was diagnosed 2 years ago. He had progressive disease despite treatment with interferon, bone marrow transplant and Glivec and also was experiencing graft *vs* host disease.
*SD score*=24. Individual SD items rated at 2 or 3: domestic chores (rated 2), work (rated 2), planning the future (rated 3), body image (rated 2), isolation (rated 3), recreation (rated 2). Non-SD item: holidays (rated 2).
*Guidance*: this is a young man with high scores ranging over a number of varied items. Mention to him that it looks like he is having quite a difficult time at present with reference to the higher scoring items. Ask what support he has and, dependent upon his response, ask him if he wishes to discuss any of these issues with the clinic staff.
*At the interview*, he described feeling very isolated as his partner was out all day working, his mother, who had been a huge support, having died a year ago and due to being generally weakened from his disease and treatment and having no car being confined to the house for long periods. This had left him feeling depressed, tearful and lacking hope. He was referred to clinical psychology for assessment and support.

*Example 3: Not socially distressed: no action indicated*
A 45-year-old woman with metastatic oesophageal cancer diagnosed 2 months ago. She started combination chemo/radiotherapy treatment and had a Hickman line.
*SD score*=8. Individual SD items rated at 2 or 3: recreation (rated 2).
*Guidance*: make a general comment on how well she appears to be doing with reference to the SDI. Unless she raises any specific issues, no other action is indicated.
*At the interview*, the patient stated that although feeling a little nauseated and tired she was managing well with wonderful support from family and friends. Her main concern was not being able to play tennis due to the Hickman line and treatment. No information, advice or referral was given.

*Example 4: Not socially distressed: action indicated*
A 62-year-old man who had completed his combination chemo/radiotherapy treatment and surgery for primary local cancer of the rectum. Attended review clinic with his wife.
*SD score*=9. Individual SD items: welfare benefits (rated 3), finances (rated 2), work (rated 2). Non-SD item: sexual matters (rated 3).
*Guidance*: this is a man with very specific high scores concerning money and work, in addition to the item on sexual matters. Ask specifically about the employment/money and sexual concerns, what support he has and, dependent upon his response, ask him if he wishes to discuss any of these issues with the clinic staff.
*At the interview*, the patient stated that he was employed but had not worked for over 6 months. His employer was ‘encouraging’ him to leave and was not sympathetic to his plight. He was receiving half his usual wage, was incurring additional expense due to his illness and was finding it hard to manage financially. Referral to social work was arranged. His sexual problems included impotence, not resolved by use of Viagra. He felt at this time that he would put up with the problem rather than pursue more help and incur more cost.
